# Emergency adrenalectomy due to acute heart failure secondary to complicated pheochromocytoma: a case report

**DOI:** 10.1186/1477-7819-9-49

**Published:** 2011-05-13

**Authors:** Carlos León Salinas, Oscar D Gómez Beltran, Juan M Sánchez-Hidalgo, Rubén Ciria Bru, Francisco J Padillo, Sebastián Rufián

**Affiliations:** 1 General and Digestive Surgery Unit -"Hospital Universitario Reina Sofía" Córdoba, Spain

## Abstract

Pheochromocytomas are catecholamine producing tumors arising mostly from chromaffin cells of the adrenal medulla. The most common clinical presentation is hypertension, mainly in the form of paroxymal episodes. Cardiovascular manifestations include malignant arrhythmia and catecholamine cardiomyopathy, mimicking acute coronary syndromes and acute heart failure.

There are reports of pheochromocytomas presenting as acute coronary syndrome and rapidly leading to cardiogenic shock; the failure of intensive medical treatment in these cases has prompted the need for emergency adrenalectomy as the only remaining option. We report on a case of complicated pheochromocytoma presenting as cardiogenic shock, in which emergency adrenalectomy was performed following a total lack of response to intensive medical treatment.

## Background

Pheochromocytomas are catecholamine-producing tumors arising mostly from chromaffin cells of the adrenal medulla.

The most common clinical presentation is hypertension, mainly in the form of paroxymal episodes. Cardiovascular manifestations include malignant arrhythmia and catecholamine cardiomyopathy, mimicking acute coronary syndromes and acute heart failure. Pheochromocytoma may constitue a clear medical emergency, and differential diagnosis poses a major challenge.

There are reports of pheochromocytomas presenting as acute coronary syndrome and rapidly leading to cardiogenic shock; the failure of intensive medical treatment in these cases has prompted the need for emergency adrenalectomy as the only remaining option.

The literature contains few papers discussing the emergency surgical treatment of pheochromocytoma. We report on a case of complicated pheochromocytoma presenting as cardiogenic shock, in which emergency adrenalectomy was performed following a total lack of response to intensive medical treatment.

## Case presentation

The patient was a 31-year-old male, with no known drug allergies. Pulmonary emphysema and an esophageal fistula had been diagnosed 7 years earlier. The patient had a recently diagnosed difficult to treat hypertension with bisoprolol and enalapril and for the last year had suffered exertional dyspnea (a recent echocardiography showed normal EF) and a number of similar episodes classed as anxiety attacks.

The patient came to the Emergency Service complaining of occipital headache, chest pain and tightness, palpitations, dyspnea and throat constriction, all of a few hours' standing; no sweating, nausea or vomiting.

Findings at physical examination were blood pressure 144/85, heart rate 98, Sat O2 100%. The patient was conscious, alert, cooperative, eupneic at rest; with hydration and perfusion satisfactory. Neurological findings were normal, without nuchal rigidity. Cardiorespiratory examination revealed rhythmic heart sounds, without murmurs and normal breath sounds. Abdomen was soft, with normal sounds, non-tender, without megalies or masses and no signs of peritonism. Lower limbs did not show edema or signs of deep-vein thrombosis. Echocardiogram revealed sinus rhythm, 70 bpm, axis normal, no repolarization defects and chest X-ray had not significant findings.

**Figure 1:**
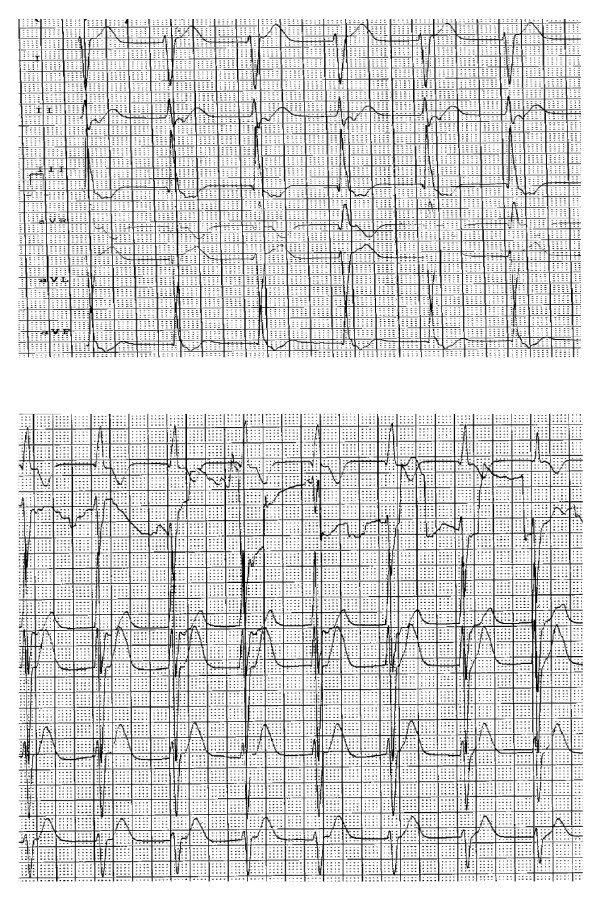
**ECG tracings during intense chest pain and hypotension episode showing a accelerated idioventricular rhythm**.

During his stay in the Emergency Unit, the patient was given sublingual lorazepam for suspected anxiety symptoms. He later displayed intense chest pain, vomiting and profuse sweating, a marked deterioration in general condition and poor peripheral perfusion. A repeat ECG revealed ST segment depression in V1, V2, V3 and V4 tracings. Troponine was elevated (table 1) Since acute coronary syndrome was suspected, electrocardiographic monitoring was accompanied by high-flow oxygen therapy, intravenous nitroglycerin perfusion, and administration of enoxaparin 80 mg, aspirin 200 mg and clopidogrel 300 mg. Peripheral perfusion remained poor, the patient complained of intense headache and blood pressure suddenly fall down without previous hypertensive episode. He also displayed rhythmic wide-complex tachycardia, which was converted to narrow-complex by intravenous administration of a bolus dose of amiodarone (2 ampoules). The patient was given 2 cc of morphine chloride and developed an accelerated idioventricular rhythm (figure 1); an emergency coronary angiography was performed with evidence of normal coronary arteries and severe depression of left ventricle function with an exertion fraction of 28%.

**Table 1:** Preoperative laboratory values of the patient

Leucocytes	20.55 × 10^3^/μl
Neutrophyls	84.9%
Hemoglobine	18.9 g/dl
Hematocrite	51.2%
Platelets	313 × 10^3^/μl
TTPa ratio	0.8
INR	1.0
Glucose	44.16 mmol/l
Sodium	142 mmol/l
Potasium	3.8 mmol/l
AST	56 UI/l
Tirosine	1.57 ng/dl
	34.6 mg/l
Creatinine	5.49 ng/ml
I - Troponine	128.48 μmol/l
RCP	

**Figure 2:**
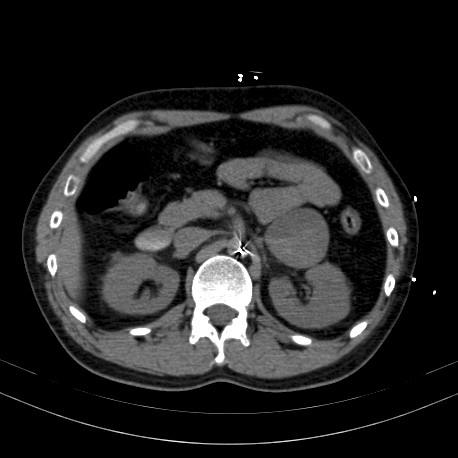
**Abdominal CT scan: a solid mass measuring 5.5 × 5 × 4 cm is visible, touching the left adrenal gland and the cauda pancreatis; the mass contains varying focal densities consistent with bleeding**.

The patient was placed in intensive care, where after 24 hours his persistently poor condition led to intubation and mechanical ventilation. On admission to intensive care, his APACHE II score was 10. He subsequently developed acute heart failure with cardiogenic shock, which failed to respond to inotropic sympathomimetic drugs. An echocardiography revealed a LVEF of 20%, prompting emergency implantation of intra-aortic balloon pump counterpulsation. A pheochromocytoma crisis was suspected because of the previous history of hypertension in a young patient and the finding of normal coronaries in the coronary angiography. An emergency abdominal CT scan revealed a left adrenal mass measuring roughly 6 cm and displaying focal necrosis (figure 2); the diagnosis was suspected complicated pheochromocytoma.

Due to hemodynamic instability and the progressive development of multiple organ failure despite intensive medical treatment, the therapeutic choice lay between extracorporeal membrane oxygenation (ECMO) and emergency vs. delayed surgery. Since no ECMO system was immediately available it was decided, following consultation with the duty surgeon and careful risk assessment (high intraoperative mortality in a patient with a life threatening condition), that emergency surgery should be performed.

**Figure 3:**
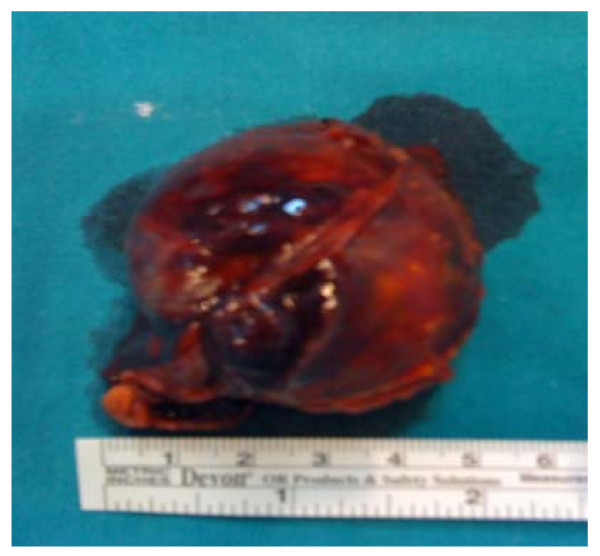
**Tumor specimen measuring 4.8 × 4.5 cm**.

Under general anesthetic, an anterior peritoneal approach through left subcostal laparotomy incision was performed; following by careful separation of surrounding structures, early ligature of the ipsilateral adrenal vein and tumor removal plus adrenalectomy (figure 3) Although the patient displayed no hypertensive crisis prior to tumor removal, he later developed hypotension which responded well to crystalloid infusion.

Histopathology was reported as a multifocal pheochromocytoma with focal necrosis and hemorrhage. Post-surgical clinical progress was good. The hydroelectrolytic balance was restored under constant monitoring. After 12 hours, the patient was extubated, the intra-aortic balloon was withdrawn and vasoactive amine thearpy was stopped. Five days after surgery, the patient was transferred to the ward, where oral tolerance therapy was started. He was then placed in the care of the Endocrinology Unit, for subsequent observation (table 2) and management.

**Table 2:** Postoperative 24-h urinary catecholamines values

Epinephrine	217.80 nmol/day (0.0 - 123)
Norephrine	233.64 nmol/day (0.0 - 504.0 9)
Dopamine	536.25 nmol/day (0.0 - 3237.0)

**Normetanephrine**	**4622.14 nmol/day (400.0 - 2424.0)**

Metanephrine	786.35 nmol/day (264.0 - 1729.0)

## Disscusion

Although pheochromocytomas are rare tumors, a relatively high prevalence (up to 0.05%) has been reported in autopsy studies, suggesting that many tumors are missed, resulting in sudden death or premature mortality [1].

Sporadic forms of pheochromocytoma are the most common (90%), and are usually diagnosed in individuals aged 40-50 years. However, hereditary forms can also occur, in association with familial syndromes (e.g. Von Hippel-Lindau syndrome, multiple endocrine neoplasia type 2, and neurofibromatosis type 1); these are usually diagnosed before age 40, and in most cases require genetic testing.

Pheochromocytoma is often referred to as the great mimic, and differential diagnosis is rendered complex by the very wide range of clinical symptoms reported [2] Whilst pheochromoytoma is found in less than 1% of patients with hypertension, between 77% and 98% of patients with pheochromocytoma are hypertensive.

Pheochromocytoma may present as a real medical emergency, mainly where there are complications [3]. Some patients present with unexplained orthostatic hypotension, which, on a background of hypertension, provides an important diagnostic clue. Hypotension may even be accompanied by shock, usually due to intravascular volume depletion, abrupt cessation of catecholamine secretion due to tumor necrosis, desensitization of adrenergic receptors, or hypocalcemia.

The serious and potentially lethal cardiovascular complications of these tumors are due to the potent effects of secreted catecholamines [4]. Pheochromocytoma may present as acute heart failure and pulmonary edema, despite coronary-artery normality [5], and may mediate acute electrocardiographic changes mimicking acute myocardial infarction (AMI) [6,7], malignant cardiac arrhythmia and even dissecting aortic aneurysm.

Other cardiovascular complications of pheochromocytoma include sudden death, heart failure due to toxic cardiomyopathy, hypertensive encephalopathy, acute cerebrovascular accident or neurogenic pulmonary edema [8-10].

The most appropriate diagnostic tests for patients with suspected pheochromocytoma remain a matter of some debate. Biochemical presentation of excessive production of catecholamines is an essential step for the diagnosis of pheochromocytoma. Traditional biochemical tests include meaurements of urinary and plasma catecholamines, urinary metanephrines (normetanephrin and metanephrine), and urinary vanillylmandelic acid (VMA); these tests have a sensitivity of over 76%. Measurements of plasma-free metanephrines (normetanephrine and metanephrine) represent a more recently available test. However, since catecholamine release is often paroxysmal, a single measurement may give a false sense of security. Sensitivity may be improved by repeating tests two or more times, and especially following a paroxysmal episode.

Other valuable diagnostic procedures include nuclear magnetic resonance imaging (which visualizes 90% of adrenal pheochromocytomas) and radio-labeled metaiodobenzylguanidine (MIBG) scanning, due to the particular affinity of this substance for chromaffin tissues. Complete abdominal CT scan may be very valuable in emergency situations; given a strong clinical suspicion, the presence of an adrenal mass is highly indicative of pheochromocytoma.

With regard to surgical treatment, elective surgery is the ideal option, accompanied by appropriate preoperative medical treatment; if the procedure is undertaken by an experienced anesthesiologist and a skilled surgeon, operative mortality is less than 1% [11]. However, in extreme conditions (e.g. shock due to a hemorrhagic necrosis or rupture of a pheochromocytoma), where hemodynamic stabilization and adequate medical pretreatment are not possible, progressive multiple organ failure may leave emergency tumor resection as the only option.

The major aim of medical pretreatment is to prevent catecholamine-induced, serious, and potentially life-threatening complications during surgery, including hypertensive crises, cardiac arrhythmias, pulmonary edema and cardiac ischemia. Traditional management strategies include the blockade of alpha-adrenoceptors; phenoxy-benzamine is mostly preferred for this purpose, since it blocks alpha-adrenoceptors non-competitively, although doxazosin is also widely used. Other alternative drugs for preoperative management are labetalol (a combined alpha- and beta-adrenoceptor blocker) or calcium-channel blockers (dihydropiridines), used either alone or in combination with adrenoceptor blockers. Metirosine (alpha-methyl-paratyrosine), which blocks catecholamine synthesis, is also occasionally used.

Medical treatment usually lasts for around 10-14 days. The alpha-adrenoceptor blocker dose is periodically increased, and a beta-adrenoceptor blocker is added after the first few days of alpha-adrenergic blockade; this treatment is particularly useful in patients with tachyarrhythmias. Additional preoperative measures include increasing salt and fluid intake (to reduce the risk of orthostatic and postoperative hypotension), maintaining blood pressure at or below 160/90 mm Hg, reducing the frequency of ventricular extrasystoles (<1 every 5 minutes) and avoiding electrocardiographic ST segment changes and T-wave inversions for one week prior to surgery [12].

Any rise in blood pressure during surgery can be controlled by bolus or by continuous infusion of phentolamine, sodium nitroprusside or nicardipine, whilst tachyarrhythmias can be treated by infusion of esmolol.

Laparoscopic removal of adrenal and extra-adrenal pheochromocytomas is now the preferred surgical technique at experienced centers, since it reduces postoperative morbidity, hospital stay, and expense compared with laparotomy [13-15], with a complication rate of <8% and a conversion rate of 5% [16]. However, open surgery may be necessary in extreme emergencies involving hemodynamic instability, where rapid action is crucial to patient survival.

After surgery, patients need to be under close surveillance for the first 24 hours, either in a recovery room or in the intensive care unit. The two major postoperative complications are hypotension and hypoglycemia. Postoperative hypotension is due to the abrupt fall in circulating catecholamines after tumor removal in the continuing presence of alpha-adrenoceptor blockade (by phenoxybenzamine). Treatment consists of fluid replacement and occasionally intravenous ephedrine. If ephedrine infusion is ineffective, vasopressin might be used. The risk of hypoglycemia is related to rebound hyperinsulinemia due to the recovery of insulin release after tumor removal.

Although there are few reports in the literature, tumor removal is known to prompt a reversal of cardiomyopathy and associated symptoms [4,17]; however, if the pheochromocytoma has remained occult over a longer period, heart transplant may be the only definitive solution.

A number of authors have reported on the use of extracorporeal membrane oxygenation (ECMO) as a rescue strategy in patients with pheochromocytoma presenting with acute cardiogenic shock not responding to intensive medical treatment, as an intermediate step prior to elective surgery [18-20].

With regard to the timing of surgery, a number of authors recommend emergency adrenalectomy whenever there is progressive deterioration of the patient's hemodynamic status or multiple organ failure despite maximal medical treatment [21,22].

## Conclusion

The initial rapid differential diagnosis in a young patient displaying clinical symptoms of acute coronary syndrome progressing to acute heart failure led to diagnostic imaging procedures which revealed a complicated adrenal pheochromocytoma; emergency surgery was seen as the only viable option, given clinical evidence of cardiogenic shock not responding to inotropic sympathomimetic drugs or emergency implantation of intra-aortic balloon pump counterpulsation. Although the first option was to continue intensive medical therapy and apply extracorporeal membrane oxygenation, the patient's declining hemodynamic status - coupled with the fact that no ECMO system was immediately available - finally led to emergency surgery within the first 24-48 hours. Good perioperative anesthesia management and a laparotomy-based surgical approach - due to the patient's unstable condition - enabled tumor removal and, within a few days, complete reversal of clinical symptoms and progressive patient recovery. Therefore, we remark the importance of emergency adrenalectomy in patients with a complicated adrenal pheochromocytoma.

## Consent

Written informed consent was obtained from the patient for publication of this case report and anny accompanying images. A copy of the written consent is available for review by Editor-in-Chief of this journal.

## Competing interests

The authors declare that they have no competing interests.

## Authors' contributions

CL and OG conceived and drafted the article. JMS and SR participated in the design of the study and review the article. FJP, RC and CL operated the patient and review the case report.
